# The Emergence of Functional Ultrasound for Noninvasive Brain–Computer Interface

**DOI:** 10.34133/research.0200

**Published:** 2023-08-15

**Authors:** Hairong Zheng, Lili Niu, Weibao Qiu, Dong Liang, Xiaojing Long, Guanglin Li, Zhiyuan Liu, Long Meng

**Affiliations:** ^1^ Institute of Biomedical and Health Engineering, Shenzhen Institutes of Advanced Technology, Chinese Academy of Sciences, Shenzhen, 518055, China.; ^2^ Shenzhen Institute of Advanced Integration Technology, Chinese Academy of Sciences and The Chinese University of Hong Kong, Shenzhen, 518055, China.

## Abstract

A noninvasive brain–computer interface is a central task in the comprehensive analysis and understanding of the brain and is an important challenge in international brain-science research. Current implanted brain–computer interfaces are cranial and invasive, which considerably limits their applications. The development of new noninvasive reading and writing technologies will advance substantial innovations and breakthroughs in the field of brain–computer interfaces. Here, we review the theory and development of the ultrasound brain functional imaging and its applications. Furthermore, we introduce latest advancements in ultrasound brain modulation and its applications in rodents, primates, and human; its mechanism and closed-loop ultrasound neuromodulation based on electroencephalograph are also presented. Finally, high-frequency acoustic noninvasive brain–computer interface is prospected based on ultrasound super-resolution imaging and acoustic tweezers.

## Introduction

Brain computer interface (BCI) technology aims to create a direct connection between brain activity and mechanical hardware or computer components. In the last decades, biomedical engineering has made substantial progress in developing neural interfaces, realizing interaction with dynamic neural systems. In 2006, Hochberg et al. [1] indicated that a 96-microelectrode array implanted in primary motor cortex could provide a valuable new neurotechnology to restore independence for paralyzed patient. Synchron Corp. developed a “stentrode”—a set of 16 electrodes to read neural activity of amyotrophic lateral sclerosis patients [2]. Willett et al. [3] proposed an intracortical BCI to decode attempted handwriting movements from neural activity in the motor cortex and translates it to text in real time. The applications of BCI spreads across multiple and diverse fields. Applications include, but are not limited to, medicine, games, education, aeronautics, and automatic drive [4–9].

**Fig. 1.**
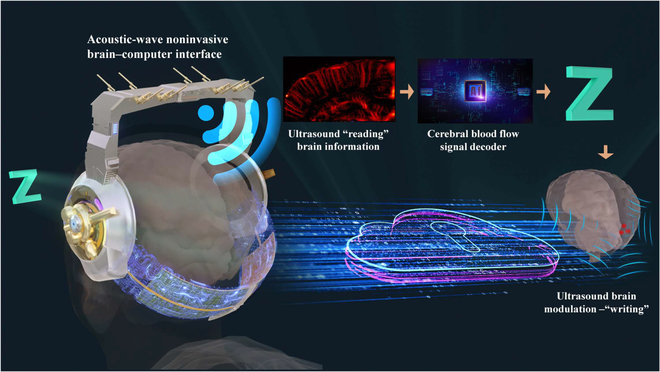
Acoustic-wave noninvasive BCI.

Remarkable progress had been made in noninvasive BCI, which had shown good development prospect. In the first successful case of noninvasive BCIs, locked-in syndrome patients controlled slow cortical potentials to select letters on a computer screen to communicate [10]. Subsequently, He et al. [11] presented and validated a framework based on noninvasive electroencephalography (EEG) to achieve the neural control of a mechanical device to track down random targets continuously. Besides bioelectrical characteristics, neural activity also produces other types of signals, such as blood flow. Functional ultrasound (fUS) imaging techniques offer a different way to monitor brain hemodynamics, read brain activity, and decode intention [12]. Transcranial focused ultrasound (tFUS) can focus on the cortices or deep nuclei to modulate activity in specific regions and affect the whole brain function using time reversal method (Fig. 1). With the advantages of better spatial resolution and safety, tFUS conforms to the development trend of neuromodulation [13,14]. Therefore, this paper reviewed the fUS imaging methods and tFUS neuromodulation technique. In the first part, the theory, methods, and applications of fUS imaging methods will be presented. Next, we describe the tFUS neuromodulation technique in detail, including the mechanism and applications in cells, rodent, primate, and human. Lastly, examples of the EEG-based ultrasound BCI is introduced. In the discussion section, we will share our viewpoints toward developing high-frequency acoustic-wave noninvasive BCI.

## Ultrasound Brain Functional Imaging–“reading”

BCI system is targeted to control computers or mechanical devices through brain activity in the absence of a peripheral nervous system. Over the past few decades, BCIs have sought to help paraplegics, consumer electronics, and other applications. Moreover, varied applications based on BCIs have been developed, such as neurorehabilitation [4], neurocommunication [6], and exoskeletons [8]. Application of BCI starts with the reading of brain information. An ideal method of brain information reading should meet the following requirements: (a) high-quality information acquisition, (b) high temporal resolution to record rapid changes in brain neurons, and (c) nonsensory or noninvasive to reduce the impact on the subjects during the reading process.

For the reading of neural signals in the brain, current BCIs gain high performance at the cost of damaging living brain tissue, which limits their applications in human neuroscience research and BCI. A noninvasive ultrasonic BCI such as fUS would open a new avenue for neuroscience research and neuroprosthetics.

At present, traditional invasive BCIs have higher spatial and temporal resolution and anti-interference performance. Noninvasive BCIs do not puncture biological tissue and are therefore more acceptable. Noninvasive reading of brain information is one of the potential directions of BCI system [15]. Currently, noninvasive BCIs utilize either the electrophysiology in the brain, via EEG or magnetoencephalography (MEG), or hemodynamic responses via functional magnetic resonance imaging (fMRI) or functional near-infrared spectroscopy (fNIRS). Noninvasive EEG-based BCIs are widely used, owing to their high temporal resolution, relatively low cost, and high portability [16]. However, EEG generally has low spatial resolution and is less sensitive to deep signals [17]. Compared to electric fields, magnetic fields are less distorted by the skull and scalp; thus, a better spatial resolution is offered by MEG compared with that provided by EEG [18]. However, the decreased signal-to-noise ratio of deep sources hinders the utility of MEG for investigations of deeper brain structures [19]. fNIRS employs near-infrared light that penetrates through the skull to detect concentration changes in oxy- and deoxygenated hemoglobin (HbO and HbR) in the brain blood supply [20]. Although fNIRS-based BCIs have gained popularity, their long response lag makes them unsuitable for real-time applications [21–23]. fMRI is a powerful method for detecting cerebral hemodynamic responses with high spatial resolution throughout the whole brain and has been used in BCI studies [24–26]. However, the response lag of fMRI-based BCI is typically 1 to 2 s, owing to its low temporal resolution [16,27].

Because it may offer information on the structure and blood flow of brain tissue noninvasively, ultrasound has become an important technique in brain science research in awake nonhuman primates and humans [28]. High-sensitivity images of cerebral blood flow can be obtained using functional and super-resolution ultrasound imaging method, which are important methods to read the brain information.

### Principle of ultrasound acquisition of brain information

The sensitivity of microvascular blood signals has been greatly enhanced, owing to ultrafast ultrasound imaging [29,30] and advances in spatiotemporal filters [31]. This enabled the detection of subtle blood variations related to neuronal activity, which eventually lead to ultrasound-based brain functional imaging [32]. Ultrasound localization microscopy (ULM) [33,34], which was recently introduced, has improved the spatial resolution of conventional flow imaging to microscopic resolutions noninvasively, even in deep brain regions [35,36]. These technological breakthroughs make ultrasound imaging a promising neuroimaging modality for BCIs.

In conventional ultrasound imaging, a typical 2-dimensional (2D) ultrasound B-mode image requires tens of ultrasound transmission and reception processes, and the maximum frame rate that can be reached is typically only a few hundred frames per second. This is far from sufficient for the high frame-rate requirements of functional imaging. An ultrafast imaging system benefits from multicore-architecture central processing units, parallel processing graphics processing units, and serialized architecture systems; thus, it can handle many channels in parallel and is able to compute a full image from only a single transmission and receipt. The multiple methods to leverage ultrafast imaging architecture, such as plane-wave imaging, represent a genuine change in the medical ultrasound paradigm. Plan-wave-based ultrafast imaging is a method to maximize ultrasound-imaging frame rates up to several tens of kilohertz, and it offers a new route for fast and transient phenomena [29].

Doppler ultrasound in rodents has been demonstrated to be substantially more sensitive to blood flow in small vessels when using ultrafast ultrasound imaging [30,37]. For example, a micro-Doppler ultrasound technique was presented by Mace [38], which substantially improved sensitivity in the detection and mapping of the cerebral blood volume (CBV) throughout the entire brain. However, blood signals, clutter signals, and electronic/thermal noise are present in ultrasound signals. A clutter filter that is usually a high-pass filter was used to reject the clutter signal and electronical/thermal noise. Each pixel's mean Doppler signal intensity and axial blood velocity information may be acquired. Noise has an impact on the blood velocity extraction; however, color Doppler is also especially sensitive to aliasing, such as an aliased signal that results in inaccurate flow direction estimation. Based on the distinct spatiotemporal coherence properties of tissue and blood movements, Demené [31] proposed clutter reduction using spatiotemporal singular value decomposition (SVD) for ultrasound data collected at a high frame rate. The customized multidimensional spatiotemporal filter based on singular-value decomposition was used to filter ultrafast imaging datasets to reduce clutter filtering and motion artifacts. Compared to the conventional clutter filter [31], this filter has a higher sensitivity for blood-flow detection.

The aforementioned ultrafast ultrasound imaging and advanced spatiotemporal filters provide ultrasound imaging with more implementation opportunities, owing to great spatiotemporal resolution (~100 μm, 1 ms) and sensitivity [38] for functional information estimation. Knowing the hemodynamic parameter being measured is crucial for functional imaging based on hemodynamic contrast. CBV and the power Doppler value are inversely related. Other techniques have also utilized functional imaging, such as CBV-weighted fMRI, which is based on the vascular injection of iron oxide particles [39]. Functional imaging has several potential applications. Understanding how the brain functions on a broad scale under normal or diseased circumstances depends on being able to track the activity of the whole brain. Power Doppler imaging, a technique used in fUS imaging, is based on an ultrafast ultrasound sequence and is sufficiently sensitive to detect blood flow in even the small vessels.

### Ultrasound-acquisition technology of cerebral blood flow and brain function information

**Fig. 2.**
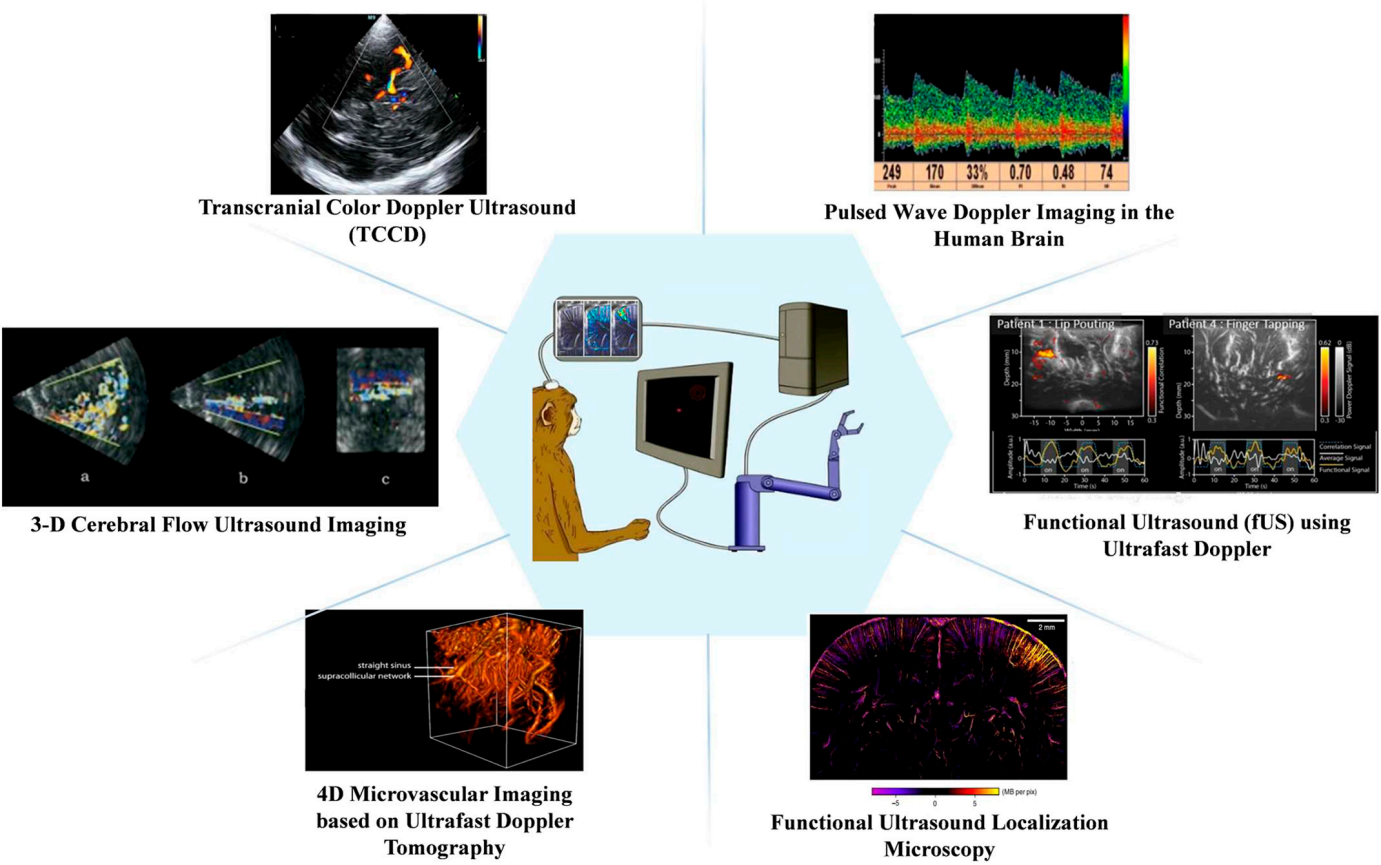
Methods of obtaining brain information using ultrasound [45–49]. Copyright (2021) IEEE; Copyright (2004) World Federation for Ultrasound in Medicine & Biology; Copyright (2015) Elsevier Inc.; Copyright (2020) The Author(s); Copyright (2022) The Author(s).

Brain-science research using ultrasound technology primarily utilizes vascular information (Fig. 2). The ultrafast ultrasound measurement of CBV can detect subtle hemodynamic changes in considerably smaller blood vessels. Similar to fMRI [40] and optical intrinsic imaging [41], fUS is based on neurovascular coupling, relying on changes in the activity of CBV in small-diameter blood vessels to detect the collection of active neurons in vivo [38]. Although CBV transmits different information from the blood, both are manifestations of neurovascular coupling [42–44], and their measurement produces indirect information about neuronal activity in the brain. Using fUS and a stimulation setup, we can investigate the feasibility of mapping in numerous animals and humans.

For animal fUS, a craniotomy was originally performed in rodents before the experiments, and the experimental animal was maintained in an anesthesia state [32]. Subsequently, a type of ultrasound-clear plastic prosthesis material, called polymethyl pentene, was applied, and it enabled cerebral imaging under the same conditions for an extended experimental period of several months [47]. Currently, a thinned skull is the main method used to acquire better fUS results [50]. Some experiments are executed while rodents are awake. Stimulation includes whisker stimulation [32], electrical stimulation [51], visual stimulation [52], and disease models, such as ischemic stroke recovery [47] and spontaneous epilepsy [32].

fUS can be used in different stimuli, typically whisker stimulation [53], followed by visual [52] and odor stimulation [53]. Whisker stimulation is an example of an activation map. Results were obtained for single-, left-, and right-whisker stimulation. Through whisker stimulation, different brain regions were observed to respond, which indicated the corresponding active regions. The Doppler signal and stimulus pattern’s correlation coefficients were used to create the maps. Finally, the results showed a substantial activation in the ventral posteromedial nucleus of the thalamus, which is connected to the thalamic cortex S1 and activated in response to tentacle stimulation. Moreover, stimulation from visual, olfactory, and pain stimuli demonstrated excellent sensitivity.

Animals provide black and white flickering stimuli with different flickering periods and luminances. The increase in CBV is observed to be related to the stimulus cycle. A similar increase in CBV was observed in the lateral geniculate nuclei and superior colliculus domains of other vision-related areas during stimulation. Moreover, fUS can accurately measure the visual-circuit activation when the left hemisphere receives visual information from the opposite visual field [52,54].

Utilizing fUS technology, we may ascertain how various scents are mapper to related regions of the anterior piriform cortex and primary olfactory bulb [53,55]. Results showed that the response of neurons and blood vessels increased nonlinearly with odor concentration. Research also shows that different smells are encoded in the main olfactory bulb for the activation of different spatial patterns. However, this encoding is in the piriform cortex, which is difficult to access in the brain structure through other imaging methods. This led to a similar diffuse activation pattern compared with other primary somatosensory cortices, and the primary visual or auditory cortex lacked stimulation.

### High-resolution microbubble localization for brain imaging

Brain imaging in small animals and clinical neonatal cases shows previously undetected blood flow, including microvascular networks or blood flow disrupted by apparent tissue or probe motion artifacts. Typically, ultrasound imaging of microvascular targets is simultaneously limited by the penetration depth and resolution. ULM was adapted from optical super-resolution imaging, such as photoactivated localization microscopy [56] and stochastic optical reconstruction microscopy [57], and it can overcome the compromise between imaging resolution and penetration depth. The ULM can achieve a spatial resolution that is 10 times better than traditional medical ultrasound imaging and provide quantitative information about blood-flow speed, owing to its ability to track moving microbubbles.

**Fig. 3.**
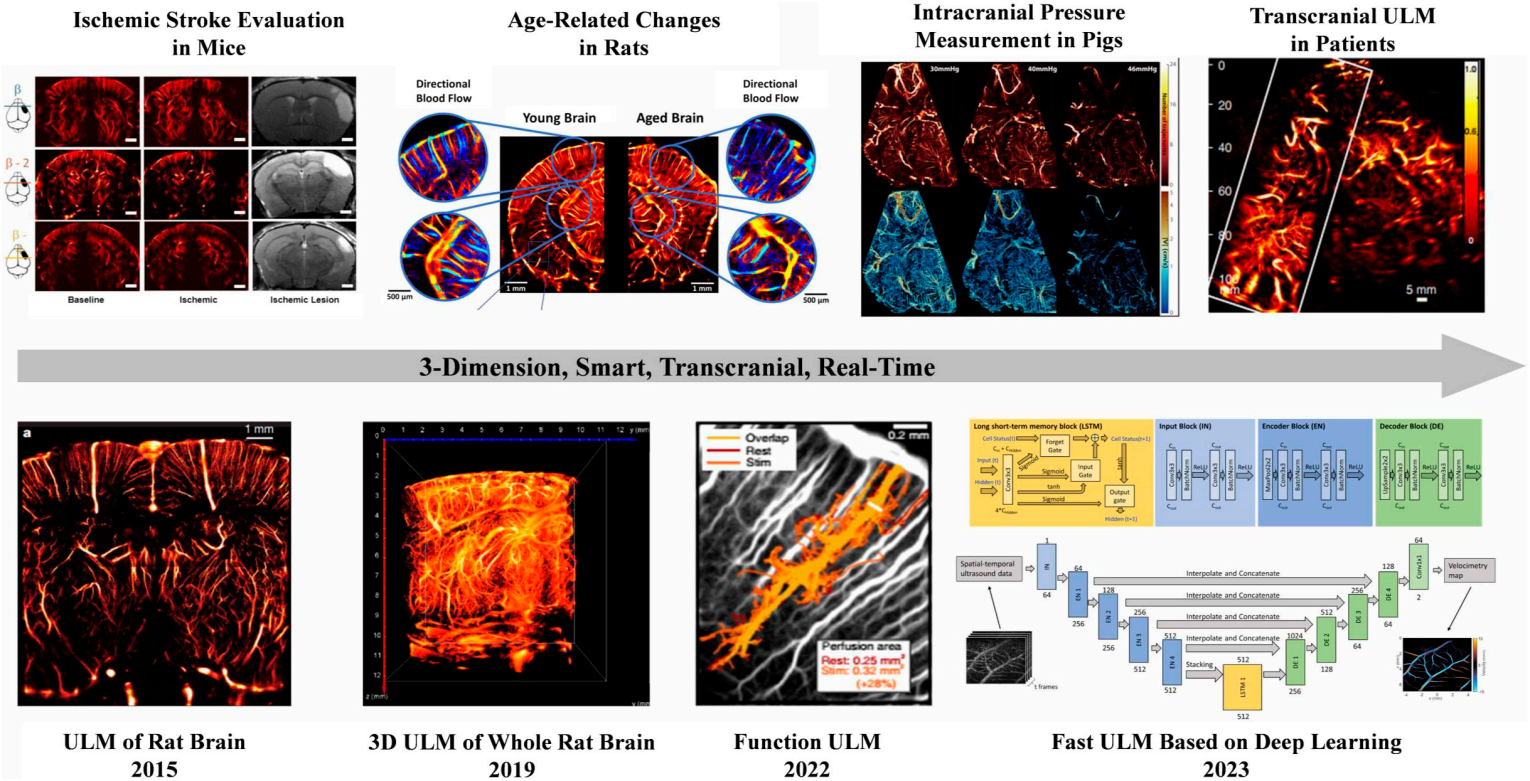
Development of ULM in brain imaging. Reproduced with permission from [35,36,49,60–65]. Copyright (2015) Springer Nature Limited; Copyright (2021) The Author(s), under exclusive license to Springer Nature Limited; Copyright (2022,2020,2022,2022,2021,2023) The Author(s).

After several initial ULM studies implemented in vitro [33,34,58,59], the basic framework of ULM was established (Fig. 3). The entire ULM procedure is as follows:

1. Long-term acquisition of contrast-enhanced imaging with a low-concentration contrast agent.
2. A filter that can separate the contrast-agent signals from tissue signals.
3. Localization step to obtain the super-resolved positions of individual microbubbles.
4. Tracking the path of microbubbles in continuous frames.
5. Accumulation tracking path and visualization of super-resolution imaging.

In 2015, Errico et al. [35] applied this technology to an in vivo setting. They demonstrated that ULM is a promising tool that can provide detailed information about microvessels in rat brains. Although this study made substantial progress, ULM remains to be optimized in all steps.

In initial research, several strategies were utilized to distinguish microbubbles, such as harmonic [33,34,59] and differential imaging. SVD was then introduced and applied to ULM to improve the process. SVD is a spatiotemporal filter used to determine the components of fast-moving bubbles in tissues [35,66]. Brown et al. [67] studied the performance of 3 bubble-detection strategies (pulse inverse, SVD, and differential imaging). Their research showed that SVD was more suitable for high-frequency ULM with fast blood flow, and the pulse-inverse method demonstrated better precision in some applications. In 2018, Song et al. [68] proposed a nonlocal mean filter to separate microbubbles from noise without any features or patterns.

The precision of localization determines the maximum resolution of ULM; thus, improving the performance of the localization approach is a key problem in ULM. The most common approach to localizing contrast agents is centroid detection using beamformed data. In most studies, microbubbles in ULM were only considered as linear point-like scatter, and algorithms such as local maxima, weight average, or gauss fit are widely used in ULM adapted from photoactivated localization microscopy. However, Christensen-Jeffries et al. [69] demonstrated localization errors owing to the nonlinear response of the contrast agent and showed that hundreds of micrometers in error are introduced when using the methods mentioned above. In 2022, Helies et al. [70] introduced performance benchmarking for localization algorithms. Several localization algorithms were compared in simulations and in vivo datasets, and the radial symmetry algorithm [71] was preferred in this study, owing to its extremely fast processing speed and moderate precision.

Because noise can be mistaken for bubbles in localization processing, tracking can act as a filter to separate microbubbles from noise and provide velocity information about microvessels. However, tracking methods still require considerable improvement. Currently, Hungarian or Kuhn–Munkres algorithms are the most frequently used approaches that focus on finding the optimal pairs of particles in continuous frames while minimizing the distance between pairing particles. This method may lead to some errors because it ignores the characteristics of blood flow and simply considers distance as the only factor. The Kalman filter, which is a widely used method, was introduced into the ULM process, and it could improve the flow-speed measurement even with a reduced number of microbubbles [72]. A dynamic method that bypassed the localization process was studied by Albert et al. [73]. Moreover, a multifeature tracking method was proposed by Yan [74] based on their study in 2005 [75]. In this work, more features were considered in the tracking processing, such as the intensity of single bubbles and Kalman motion model.

Motion is unavoidable in acquisition processing and greatly affects ULM quality. Thus, motion correction is essential for ULM in vivo. Hingot et al. [76] designed a simple subwavelength motion-correction method using the cross-correction of 2 frames; however, this method is only helpful when only planar motion exists. Harput [77] introduced a 2-stage motion-correction algorithm adapted from MRI, which was capable of nonrigid motion, but was time-consuming. One of the practical strategies to reduce the influence of motion caused by the breath or heartbeat is to use electrocardiogram gating to ensure that acquisition is only performed during the rest period of the breath or heartbeat [78]. Specifically, only planar motion must be considered for applications in brain imaging.

However, the methods mentioned above cannot correct out-of-plane motion. Three-dimensional (3D) ULM is considered an important developing area of ULM that can obtain full information about the region of interest without out-of-plane motion. Moreover, 3D ULM has already been implemented in vitro using different imaging strategies, such as sparse arrays [79], row–column arrays [80], and synchronized systems [81]. Additionally, several encouraging in vivo results have been reported using a full-matrix array [82,83] with a full-sampled system or multiplexer [84].

A long acquisition time still limits the clinical applications of 2D or 3D ULM because the model-based framework fails to localize high-concentration microbubbles, which results in a long acquisition time to obtain sufficient tracks. In contrast to traditional algorithms, deep learning is expected to reduce the required processing time. A deep learning framework was proposed by Milecki et al. [85] that could obtain a super-resolution image at high microbubble concentrations where the traditional method failed. In 2021, Li et al. [86] presented a self-supervised deep learning network that could improve the performance of microbubble localization at high microbubble concentrations without using the ground truth. These works improved the imaging speed but could not measure the blood-flow velocity. Chen [87] designed a long short-term memory neural network to localize and track a moving bubble at high concentrations. Their work showed that the network could substantially improve the performance of ULM, and both the acquisition and processing times were considerably reduced.

In brain-imaging applications, traditional brain-ultrasound imaging is influenced by the skull. However, ULM can be used to perform transcranial imaging with an intact skull. Although attenuation and aberration of the skull still degraded the ULM performance, this study proposed an effective skull-aberration correction that calculated the aberration delay [88–90], which has exhibited good performance in patients’ brains [36].

### Challenge of decoding brain information using ultrasound imaging

As a brain information reading method, fUS has the multiple advantages. Because ultrasound is more portable, it allows for long-time, continuous measurements of free-moving animals [91] compared to methods such as fMRI. Besides, fUS also provides more spatial information than EEG, including information on global blood flow and structures deep in the brain [92], which makes fUS a very promising brain reading tool in BCI applications. In particular, the recently proposed ULM or super-resolution ultrasound leverages ultrafast ultrasound and microbubble localization technology to greatly increase the resolution of cerebral blood-flow imaging up to tens of microns. It breaks through the diffraction limit of brain ultrasound and opens up a new field for fUS brain imaging. However, there are a number of challenges in trying to decode brain information using US:

First, the global blood-flow signal changes more slowly than the EEG signal [93], and it is unclear whether accurate decoding of brain information can be achieved. Although numbers of publications mentioned above have recorded images of CBV changes of the brain in the presence of stimuli. However, these works only demonstrated in the certain brain regions where blood-flow changes are highly correlated with stimulus signals rather than the actual physiological state of the brain regions. The link between fUS signal and the physiological state of the brain needs to be further verified.

Compared to EEG, fUS can provide more information, such as structural and blood-flow changes deep in the brain instead of electrophysiological signals in highly specialized brain regions, which also places greater demands on the decoding algorithm. How to accurately and fully utilize the rich temporal and spatial information of fUS is very challenging and meaningful study. Berthon et al. [94] used artificial neural network to decode the global blood-flow information obtained from fUS to decode the behavior of the brain, and their network was able to accurately predict the movement or resting of rats, demonstrating the potential of artificial neural network in BCI based on fUS.

Finally, fUS is a pixel/spatial location-based means of reading brain information. The differences in brain anatomy between the 2 subjects, or different relative positions of the probes during the 2 acquisitions, can result in pixels not being matched to the corresponding anatomical regions, thus affecting decoding outcome by the decoder. A perfect match between the 2 sets of data is required to achieve the correct decoding and encoding of brain information [94]. Thanks to the 3D ultrasound, whole-brain imaging can be achieved to reduce the impact of differences in brain slice acquisition, but the huge data volume of 3D ultrasound may limit the BCI's online application.

## Ultrasound Brain Modulation and Possible Writing

### Ultrasound neuromodulation and its applications

**Fig. 4.**
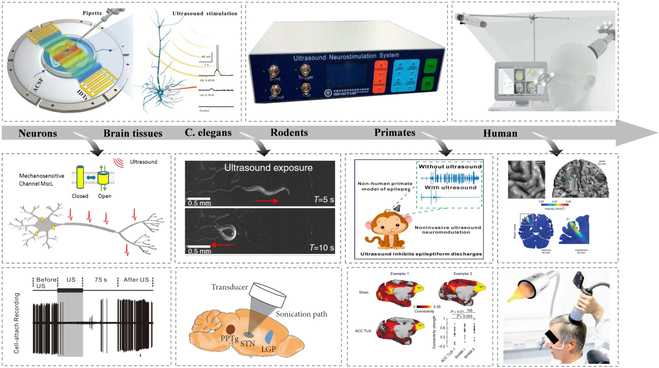
Ultrasound neuromodulation has been applied in many animal models and humans. Reproduced with permission from [13, 104–112]. Copyright (2014) Springer Nature America, Inc.; Copyright (2018) WILEY-VCH Verlag GmbH & Co. KGaA, Weinheim; Copyright (2020) The Author(s); Copyright (2018) American Chemical Society; Copyright (2015,2020,2020,2020,2019,2019,2019) The Author(s).

Ultrasound is a sound wave (>20 kHz) beyond the range of human hearing. As early as the 19th century, in the process of studying the mutual conversion of mechanical and electric waves, electric current was applied to piezoelectric crystals to convert energy into mechanical motion to generate sound waves [95]. Ultrasound neuromodulation technology is a novel neuromodulation method that simultaneously combines noninvasiveness and high precision [96] with high spatial resolution and depth. Ultrasound neuromodulation technology uses ultrasound waves emitted by ultrasonic transducers to act on brain nuclei through the skull, muscles, and other tissues to regulate neural activity [97]. In 1955, Fry et al. [98] observed that ultrasound stimulation of a cat’s lateral geniculate nucleus reversibly suppressed evoked potentials in the primary visual cortex. In the past 20 years, ultrasonic neuromodulation technology has entered the era of “big explosion”. In 2005, Huang et al. [99] applied ultrasound to the isolated sciatic nerve of a bullfrog and observed that ultrasound could affect the compound action potential and conduction velocity of the nerve. In 2008, Tyler et al. [100] demonstrated low-intensity ultrasound-induced neural activity in mouse hippocampal slices and proposed a possible mechanism by which ultrasound affects voltage-gated sodium and calcium channels. In 2010, they demonstrated the neuromodulation effect of low-intensity ultrasound in small animals for the first time [101]. In 2011, Yoo [102] showed that ultrasound is a dual-modal neuromodulation technology that can selectively activate or inhibit brain activity in their study of ultrasound regulation of the somatomotor area and visual area of rabbits. In 2013, Thomas et al. [103] used ultrasound to change the direction of eye movements in monkeys, thereby demonstrating that ultrasound can modulate neural activity in nonhuman primates. In 2014, Wynn et al. [13] applied low-intensity ultrasound directly to the primary somatosensory cortex of the human brain, which changed the ability of the human brain to distinguish touch and realized the application of ultrasound neuromodulation in the human body. Over several years, the neuromodulatory effects of ultrasound have been validated in isolated tissues, rodents, nonhuman primates, and human clinical studies, as shown in Fig. 4. In addition, ultrasound neuromodulation technology can change and modulate the physiological activities of the normal brain, and it has potential applications in the neuromodulation treatment of neurological diseases.

Regarding cerebrovascular-related diseases, Furuhata et al. [113] observed that in a rat acute-stroke model, low-intensity ultrasound enhanced the thrombolytic effect of systemic tPA administration, thereby substantially reducing cerebral infarct volume and improving neurological status. Tong et al. [114,115] showed that ultrasound stimulation of the ischemic-core region immediately after ischemic stroke in rats alleviated ischemic symptoms after brain injury. In addition, they observed that ultrasound stimulation can reduce the symptoms of focal ischemia in rats before inducing ischemic stroke. Liu et al. [116,117] observed that the early treating time window is the key to ultrasound's protective effect on acute ischemic stroke.

Studies have shown that chronic ultrasound stimulation can effectively and safely improve depression-like behavior in rats with neuropsychiatric diseases. The potential mechanisms may be associated with the activation of the brain-derived neurotrophic factor/extracellular regulated protein kinases/mammalian target of rapamycin signaling pathway in the prefrontal cortex (PFC) via ultrasound, which increases the release of the brain-derived neurotrophic factor [118,119]. Niu et al. [120] showed that the reduction of inflammatory cytokine expression in the PFC by ultrasound is also an important mechanism by which ultrasound improves depression-like behavior. In addition, multiple clinical studies have demonstrated that ultrasound stimulation of the PFC can substantially improve the mood of healthy volunteers and alleviate depressive symptoms in Alzheimer’s disease (AD) patients [121,122].

Parkinson's disease (PD) is a typical neurodegenerative disease, and the prevalence of this condition increases with age. Pharmacological treatments are typically used as regular treatment for PD. For patients who are unable to tolerate the side effects of pharmacy or show no response to it, surgical treatments offer another choice. Zheng et al. [110,123,124] indicated that ultrasound stimulation of the motor cortex or subthalamic nucleus could alleviate the motor symptoms of mice with PD by inhibiting neuroinflammation, and the treatment had an effect on dopamine. Neurons are neuroprotective. Mason et al. [125] showed that ultrasound stimulation could increase dopamine content and improve exercise capacity in PD mouse models. Li et al. [126] showed that ultrasound neuromodulation can substantially reduce related EEG activity in a mouse model of PD. Low-intensity pulsed ultrasound stimulation may antagonize PD by enhancing glial-cell-line-derived neurotrophic factor levels and inhibiting the inflammatory response in the brain [127,128].

Mourad et al. [129] showed that ultrasound stimulation of the hippocampus of AD model mice can reduce amyloid aggregation. Leinenga et al. [130] removed Aβ protein deposits and restored memory in AD mice using scanning ultrasound. Niu et al. [131] observed that ultrasound stimulation could delay telomere shortening in cortical and myocardial tissues and improve spatial cognition and learning in AD mouse models. Beisteiner et al. [122,132,133] reported that a single ultrashort ultrasound pulse acting on the brain's memory network substantially improved neuropsychological scores in patients, and the improvement persisted for up to 3 months. The beneficial effects of ultrasound in patients with AD can be attributed to the improvement in cortical atrophy via ultrasound modulation and the regulation of brain neural-network function by inducing neuroplastic changes [133–135].

Drug addiction is a psychiatric disorder that affects the limbic reward circuit. Deep brain stimulation is a noninvasive modulation method that is used to improve symptoms. Recently, Niu et al. [136] observed that ultrasonic stimulation can effectively and rapidly reduce the behavioral preference induced by morphine, and its persistent effect can change relapse behavior after withdrawal in mice. This suggests the considerable potential of ultrasound neuromodulation technology for drug-addiction treatment.

Neuropathic pain, such as a chronic pain disorder, is typically caused by nerve damage [137,138]. Pilitsis et al. [139] showed that ultrasound stimulation of the rat dorsal root ganglia increased mechanical and thermal sensory thresholds, and this improvement was more substantial in female rats [140]. They also demonstrated that ultrasound can alter pain behavior and allodynia in a porcine peroneal nerve-injury model [141]. This may be related to the improvement in the inflammatory response of the dorsal root ganglia via ultrasound stimulation [142]. In addition to acting on the peripheral nerves, ultrasound can also modulate the central brain nuclei to treat pain. Niu et al. [143] observed that ultrasound stimulation of the anterior cingulate gyrus can effectively relieve mechanical neuropathic pain. Zhu et al. [144] used the ultrasound stimulation of the periaqueductal gray to effectively inhibit formalin-induced nociception.

At the rodent level, epilepsy may occur in all age groups, and it is one of the most common neurological disorders. Ultrasound neuromodulation has reduced abnormal electrical discharges in epileptic brain regions [145–148] and improved behavioral abnormalities in chronic epilepsy [149]. Recently, Niu et al. [150] indicated that ultrasound has a neuroprotective effect, thereby inhibiting neural apoptosis in epileptic mice and improving epilepsy. In addition, Zheng et al. [108,109] showed that ultrasonic neuromodulation technology can safely and effectively regulate the electrical activity of neurons in the brain, inhibit the abnormal discharge of neurons in the brain tissue of epilepsy patients, and improve the behavior of epileptic monkeys. Several clinical studies on epilepsy patients have proven that ultrasound neuromodulation can safely and effectively suppress epileptic discharges in patients [14,151].

## Direct Ultrasonic Modulation of Neural Activity and Sonogenetics

Manipulating local or global neural activity using different external physical stimuli, such as electrical, magnetic, optical, or acoustic stimulation, has provided several avenues for both basic research and clinical therapy. However, diverse modalities and techniques are limited by their inherent physical or biological drawbacks, including invasiveness, spatiotemporal resolution, and stimulus depth. An emerging approach that uses ultrasonic waves to control neural activity has sparked wide attention because ultrasound wave can noninvasively stimulate deep brain structures through an intact skull with millimeter-sized dynamic focal spots and high spatiotemporal precision. Scientific research on evaluating ultrasonic neuroregulatory effects began decades ago [98], and a number of studies that applied ultrasonic waves to excite or reversibly suppress neural activity have emerged in recent years for cultured cells, brain slices, various animals, or humans with different acoustic parameters and focused targeting. The biophysical effects of ultrasonic wave interactions with biological tissues are complex and include mechanical force, heating, cavitation, and off-target auditory effects [95,152,153]. The underlying mechanisms of ultrasonic neuromodulation are not well understood; however, a number of studies have hypothesized that ultrasonic waves may mediate neural activity through mechanosensitive ion channels. Hence, we have summarized the mechanosensitive ion channels for direct ultrasonic stimulation in unmodified cells and the exogenous expression of mechanosensitive ion channels for sonogenetic control in modified cells.

Ion channels are a class of proteins embedded within cellular membranes that allow different ions to pass through the channel pore and act as molecular switches to control neural activity. In 2008, Tyler et al. [100] illustrated that ultrasound can stimulate the neural activity of hippocampal slice cultures and ex vivo mouse brains by activating voltage-gated sodium and calcium channels. Our previous results have also shown that ultrasound can increase transmembrane sodium and potassium ion currents and the kinetics of sodium channels in hippocampal slices using patch-clamp recording in vitro [104,154]. Kubanek et al. [155] expressed two pore domain potassium channels in *Xenopus* oocyte system, including TREK-1, TREK-2, TRAAK, and NaV1.5, and showed that the current regulation of ultrasonic convection through ion channels can reach 23% on average. Using single-channel recording, Sorum et al. [100,156,157] further confirmed that the ultrasonic activation of the mechanical sensitive K^+^ channel TRAAK has submillisecond kinetics, which is comparable to the standard mechanical activation. Ultrasound-induced intracellular calcium ion (Ca^2+^) transients through Ca^2+^ channels have also been reported in several cell types. Burks et al. [156] indicated that ultrasound activates a Na^+^-containing transient-receptor-potential-channel-1 current upstream of voltage-gated Ca^2+^ channels in the kidney and skeletal muscle. Yoo et al. [157] also suggested that ultrasound excites mouse primary cortical neurons in vitro through specific Ca^2+^-selective mechanosensitive ion channels. Studies by Kubanek et al. and Zhou et al. [158,159] confirmed that ultrasound elicits behavioral responses in *Caenorhabditis elegans* through the MEC-4 channel, which is a DEG/ENaC/ASIC ion channel required for touch sensation. Piezo channels, including Piezo 1 and Piezo 2, are among the few eukaryotic channels that can be directly activated by ultrasonic waves in cultured cells or mammalian peripheral neurons [160,161]. Interestingly, Oh et al. [162] reported that TRPA1 channels in astrocytes act as unique sensors for ultrasound neuromodulation, and glutamate-releasing Best1 mediates of the glia–neuron interaction.

**Fig. 5.**
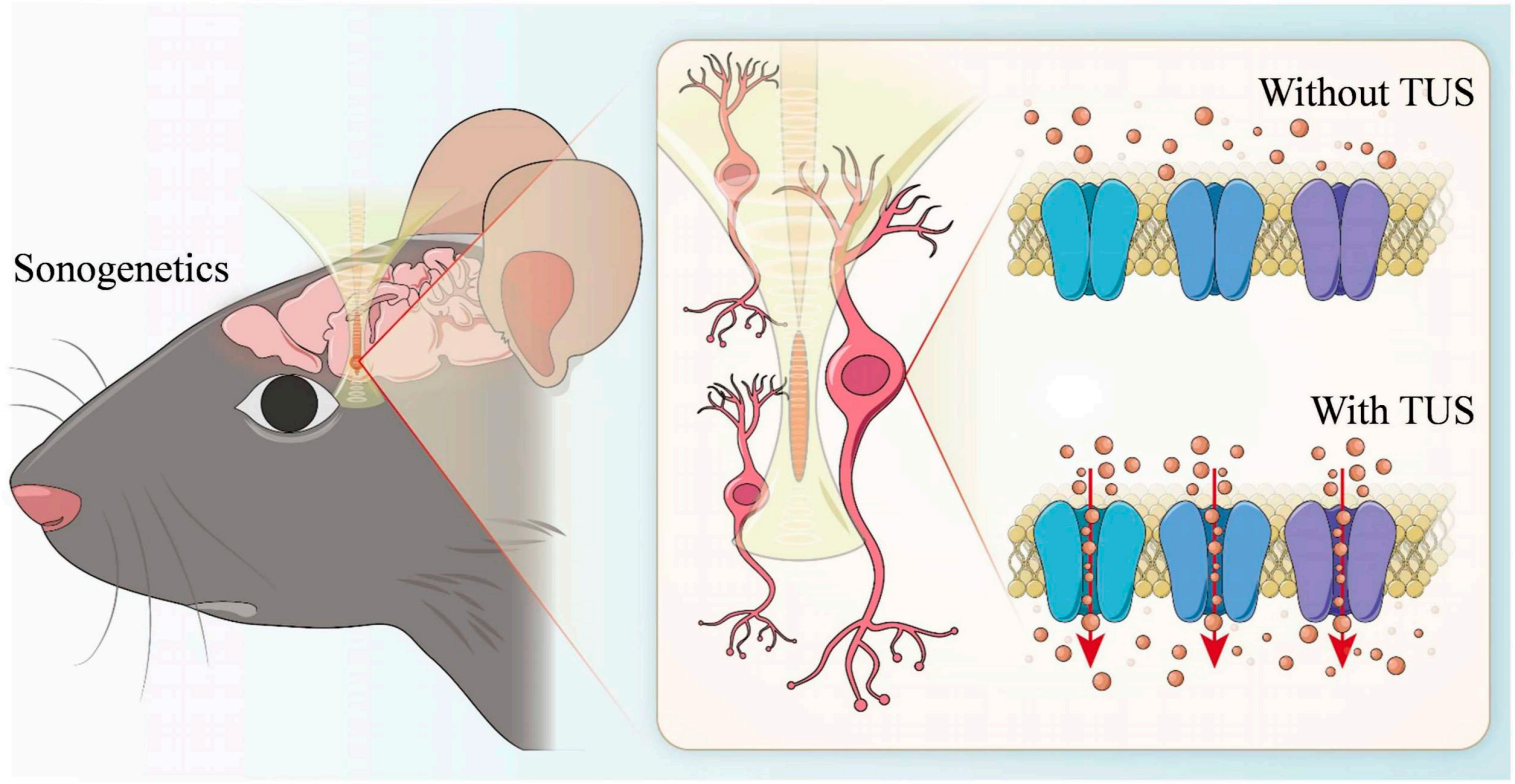
Direct ultrasound stimulation and sonogenetics.

Similar to optogenetics or chemogenetics, which make use of transgenic ion channels that target specific cellular populations to control neural activity, a new technology called “sonogenetics” has been developed, which utilizes ultrasound to noninvasively control neural activity through engineered specific cells with ultrasound-responsive proteins (Fig. 5). The concept of “sonogenetics” was proposed in 2015; Ibsen et al. [107] used microbubbles to overexpress TRP-4 mechanosensitive ion channels in *Caenorhabditis elegans* to trigger reversible behavioral responses. Following this pioneering work, a series of ultrasound-responsive proteins has been reported to sensitize cells to acoustic stimuli. In 2018, Ye et al. [106] expressed the *Escherichia coli* mechanosensitive channel of large conductance (MscL) and its gain-of-function mutation, I92L, in rat hippocampal neurons in primary culture and showed that the channel could be activated by ultrasound. Qiu et al. [163] also proved that ultrasound can induce Ca^2+^ influx and neuron activation in vitro and evoke electromyography (EMG) response in targeted cells by activating heterologous mechanical sensitive channels (Mscl-G22S). In 2019, Qiu et al. [164] showed that ultrasound alone could activate the heterologous and endogenous mouse piezo-type mechanosensitive ion channel component 1 (Piezo1), which initiates calcium influx and increases nuclear c-Fos expression in primary cortical neurons. In the same year, Huang et al. [165] reported an ultrasound-responsive engineered auditory-sensing protein, prestin (N7T, N308S), which has the ability to sense ultrasound stimuli in vitro and in vivo. The same research group further targeted this ultrasound-responsive protein in the dopaminergic neurons of the substantia nigra in mice with PD and indicated that ultrasound ameliorated dopaminergic neurodegeneration and mitigated PD symptoms [166]. In 2022, Duque et al. [167] identified human TRPA1 as a candidate ultrasound-responsive protein that potentiates ultrasound-evoked responses in primary neurons in vitro and leads to c-Fos expression and contralateral-limb responses in vivo. In addition to mechanical sonogenetics, Yang et al. [168] developed sonothermogenetics to selectively activate neurons through expressing thermosensitive ion-channel transient receptor potential vanilloid 1 in the mouse brain in vivo.

The mechanisms proposed to explain the ultrasound neuromodulation effects are based on multiple hypothesis on how ultrasound interferes with depolarization through mechanical deformation of the cell membrane. In addition, experimental evidences have shown that ultrasound can activate mechanosensitive ion channels in neurons [100,106,161,164] and other brain cell types like astrocytes [162], providing additional avenues for ultrasound neuromodulation to interfere with the membrane potential.

While the lack of a complete understanding of the ultrasound neuromodulation mechanisms does not currently impede reaping potential benefits in a more application-driven context (e.g., neurodegenerative and neuropsychiatric disorders) [132,169], ultrasound BCI offers a new way to understand mechanisms.

Two recent studies argued that ultrasound neuromodulation requires auditory pathway activation in rodents [153,170]. However, recent work by Niu et al. [171] using chemically deafened rodent models showed that the ultrasound brain modulation is confined by localized response without involving auditory networks. Our previous study has indicated that ultrasound-induced behavioral changes are attributed to direct activation to the related brain region rather than to the involvement of the auditory pathway [172]. In addition, hearing range of mouse is from 2.3 to 85.5 kHz [173], which is far below the fundamental frequency of ultrasound neuromodulation. Ultrasound-evoked auditory activation is influenced by acoustic parameters, particularly pulse repetition frequency (PRF) [174], which can be inhibited by the waveform shape and special acoustic masks [174,175].

## Ultrasound BCI-Preliminary Studies Based on EEG

**Fig. 6.**
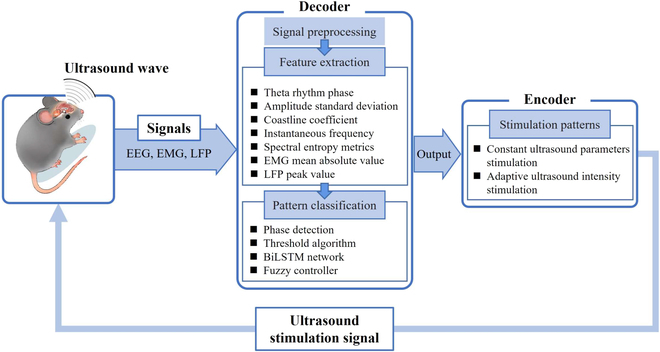
Closed-loop ultrasound BCI based on EEG.

The demand for ultrasound stimulation systems has increased with the development and improvement of ultrasound neuromodulation techniques. Most ultrasound neuromodulation methods mentioned in the previous section use open-loop stimulation, which generates stimulus signals according to a preset schedule, but the stimulation cannot be automatically adjusted according to the changes of physiological signals. Thus, a closed-loop brain neuromodulation system combining existing physical neuromodulation technologies, such as optogenetics, deep brain stimulation [176], transcranial electrical stimulation [177], and transcranial magnetic stimulation has been developed [178]. There have also been recent reports on closed-loop ultrasound neuromodulation systems, owing to the advantages of closed-loop brain stimulation [179–181] (Fig. 6).

The design of a closed-loop brain-stimulation system requires a decoder and an encoder, where the decoder is used to decode the biological signals into the stimulus intent, and the encoder is used to convert the stimulus intent into a stimulus signal. Yang et al. [179] designed a closed-loop transcranial ultrasound stimulation (CLTUS) system that included decoding the local field potential (LFP) and encoding the level signals. The decoder design included 2 parts of experiments for the modulation and suppression of temporal lobe epilepsy (TLE) seizures. An experiment was performed to verify whether CLTUS can detect the phase of the theta rhythm in real time and stimulated mouse CA1 in a specific phase. The decoder predicts the next peak or trough by analyzing the data. The specific implementation first used the Hamming window to devise a band-pass filter to obtain theta rhythm signals. Then, setting the period threshold, calculating the amplitude of the theta rhythm to determine the location of all the maximum and minimum values. Finally, the next peak or trough moment was predicted based on the calculated average period, peak-to-peak value, and time delay between the signal acquisition and theta rhythm determination. Once the phase of a specific theta rhythm was predicted, it entered the encoding process, which means that the computer transmitted a transistor–transistor logic high-level signal to initiate an ultrasound stimulation system consisting of a function generator, power amplifier, and ultrasound transducer. The results showed that the LFP amplitude, the theta rhythm amplitude, and power intensity at both specific phases increased substantially after CLTUS, while the relative power of the theta rhythm decreased. Furthermore, there was no correlation between the stimulation effect and the specific phase (peak or trough) of the theta rhythm. The system has been studied in a mouse model of TLE to confirm its ability to deal promptly and successfully with neurological disorders that are accompanied by abnormal neuronal firing. In this part of the study, the standard deviation of the amplitude and coastline coefficient of the LFP from CA1 were calculated as eigenvalues to detect seizures. Seizure status was determined when the standard deviation of the data amplitude was more than 3 times the baseline value, and the coastline coefficient was more than twice the baseline value. When the conditions for the recognition of the seizure status are met, the computer also sends a high-level transistor–transistor logic signal to activate the ultrasound stimulation system to stimulate the hippocampal region of the mice. CLTUS system can be used to monitor epilepsy in real time and provide ultrasound stimulation according to the monitoring results. The seizure latency was longer, and the seizure duration was reduced obviously in CLTUS-treated TLE model mice. In the experiment, an ultrasound transducer with a fundamental frequency of 500 kHz was used. The PRF was 1 kHz, and the duty cycle was 40%. For the experiment on the modulation of the TLE, the alternate parameters were 500 kHz, 500 Hz, and 5%, respectively. All the experiments used an *I*_SPPA_ value of 1.75 W/cm^2^.

Recently, artificial intelligence and other methods have proved to be powerful tools for decoding brain signals. Zhong et al. [180] conceived a closed-loop ultrasound deep brain stimulation system based on a deep learning decoder, which can focus ultrasound on the hippocampus through a wearable transducer, proving its good efficacy. The 1-s captured EEG signal were first obtained by a 1- to 40-Hz finite impulse response band-pass filter. Subsequently, extracting the instantaneous frequency and spectral entropy metrics as the time-frequency and nonlinear features of the preprocessed EEG signal. Finally, the above obtained feature values are combined into coupled feature sequences and brought into the bidirectional long short-term memory (BiLSTM) network architecture for training, which consisted of a sequence input layer, 2 BiLSTM layers with a dropout layer in between, a size 2 fully connected layer, a softmax layer, and a classification output layer. The BiLSTM model was obtained using the aforementioned training. The EEG signals were manually examined to correctly classify the training dataset. The accuracy of the BiLSTM model was 81.33%. Then, continuous timely monitored EEG were sent into the optimal BiLSTM model by close-loop wearable ultrasound stimulation system, and the hippocampus was stimulated with a 10-min activation of the ultrasound stimulation system when a 10-s sustained seizure was monitored (fundamental frequency = 800 kHz, PRF = 1 kHz, duty cycle = 40%, tone burst duration = 0.4 ms, interstimulus interval = 3.6 s). The closed-loop wearable ultrasound deep brain stimulation system constructed using BiLSTM could successfully supervise epileptic seizures automatically. The power spectral density of EEG signals was substantially reduced, the duration of epileptic seizures was substantially shortened, and more early epileptic mice survived. In addition, an LSTM model was constructed and compared with the BiLSTM model in terms of classification accuracy; the performance of the network was confirmed through 5 cross-validations. The consequences show that BiLSTM network performs better than LSTM network in classification performance.

Based on the CLTUS system, Yuan et al. [181] added a fuzzy algorithm to the control program to achieve timely and accurate control of the limb movement and nervous system of mice. The target of the ultrasound stimulation in this study was the motor cortex. The mathematical basis of the CLTUS system is a model between the mean absolute value (MAV) of electromyogram (EMG), peak value (PV) of LFP, and ultrasonic intensity. The EMG and LFP signals were obtained by artificially changing the PV of the generator output signal from 350 to 750 mVpp (corresponding to the sound pressure value of 0.21 to 0.78 MPa) at 50-mVpp intervals under the open-loop stimulus condition. Once the EMG and LFP signals with the length of 90 s were collected through the electrode, they were first passed through notch filter to remove 50-Hz ac power interference, and at the same time the baseline drift got removed by passing through an adaptive high-pass filter. Then, the EMG signal is filtered by 300- to 1k-Hz bandpass, rectified, and passed Gaussian filtering to become the preprocessed EMG. The LFP signal is filtered by 4- to 200-Hz bandpass, and the analysis signal of LFP obtained by Hilbert transform can be used to calculate PV and LFP envelope after modulo. Following processing by back propagation neural network, the discrete relative datasets of EMG MAV, and LFP PV and different ultrasound intensity are obtained. The network consists of 3 hidden layers with 10, 10, and 2 neurons in each layer, based on extensive open-loop experimental data. The equations that described the EMG and ultrasound intensity, LFP and ultrasound intensity, and corresponding parameters were obtained using least squares fitting. The input of the back propagation neural network was ultrasound intensity, and the output was TUS-induced EMG MAV or LFP PV with an accuracy of 0.001. A 2D fuzzy controller is employed to regulate the ultrasound intensity immediately, which had 2 input ports, one for the error and one for the relative error between MAV or PV corresponding to EMG or LFP respectively. The closed-loop modulation of animal EMG and LFP is realized through the intensity regulation factor output by the controller. After the fuzzy-controlled closed-loop TUS system was established, the controllers were designed using a proportional integral derivative algorithm and an immune feedback algorithm, and the control effects of the 3 controllers were compared using simulations and practices. The performance of fuzzy controller is better than the other 2 methods; not only the relative error is substantially less than proportional integral derivative and immune feedback control but also the output value is in line with the expected value. It is proved that the fuzzy controller can be used to control EMG and LFP in mice. The closed-loop TUS system using the fuzzy controller enabled a more accurate and rapid tracking of the desired value. These results show that the fuzzy-control-based CLTUS system is capable of stable in vivo CLTUS.

Research on closed-loop ultrasound neuromodulation is in its initial stage. However, this technology will be developed and progressed further because it meets the needs of timeliness and can provide further enhancement of neuromodulation and disease therapy. Ultrasound neuromodulation with artificial structure can achieve multi-target and dynamic stimulation with high spatiotemporal resolution in brain, thus it may be used to restore useful visual or auditory functions to blind or deaf people and to allow more efficiently transform information within other cortical prosthetic applications. Currently, the biological signals used for closed-loop ultrasound neuromodulation are EMG, LFP, and EEG, and it is believed that more biological signals will be used for decoding in the future, such as fMRI, positron emission tomography, and ultrasound brain functional imaging.

**Fig. 7.**
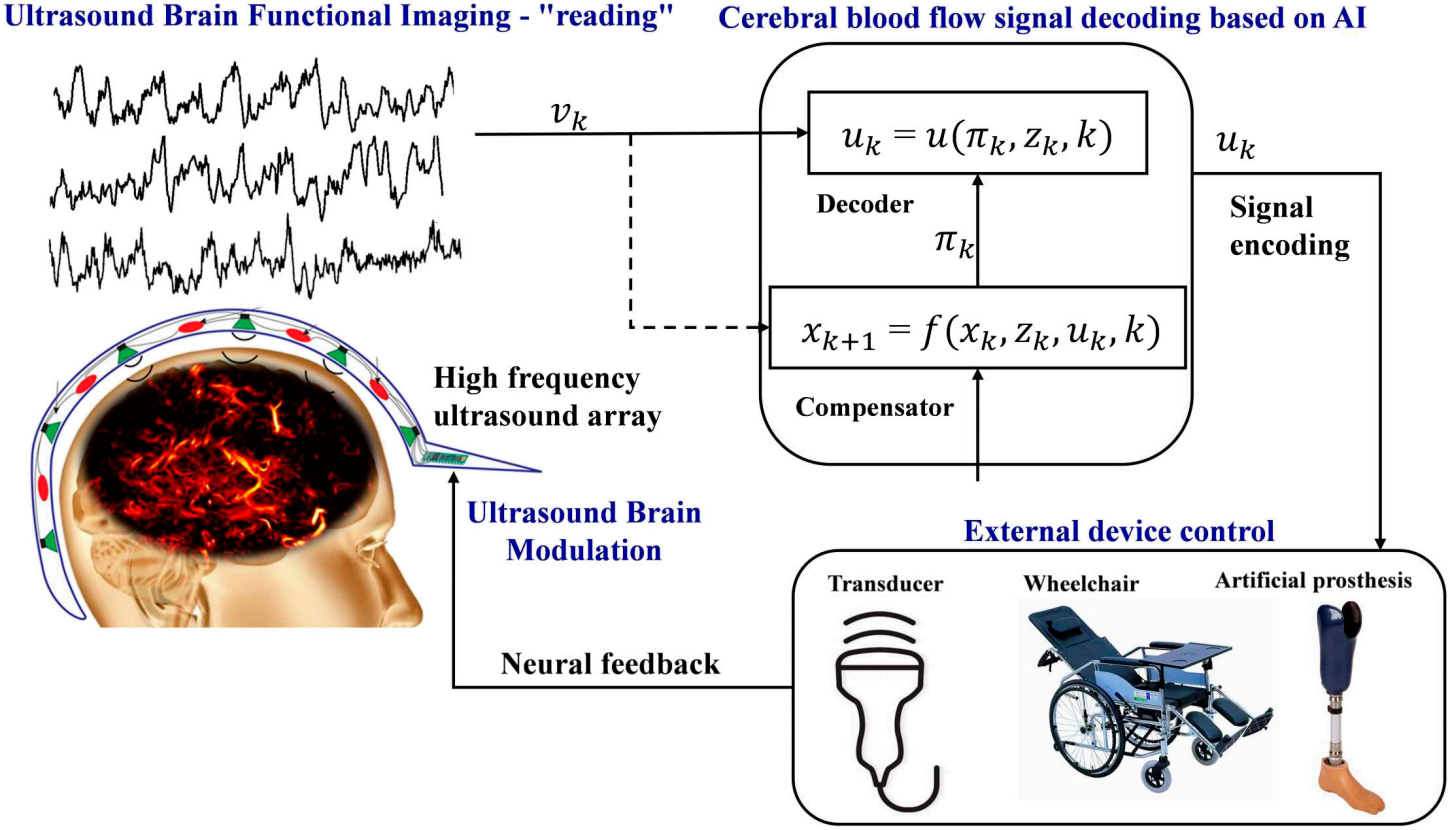
Functional ultrasound for noninvasive BCI. Reproduced with permission from [36]. Copyright (2021) The Author(s), under exclusive license to Springer Nature Limited.

Therefore, we propose fUS brain imaging to noninvasively “read” neural activity in the brain and apply ultrasound radiation to open mechanosensitive ion channels to precisely “write” neural information, thereby realizing a noninvasive ultrasound closed-loop brain–machine interface, as shown in Fig. 7. First, combined with ultrasound contrast microbubbles, a high-sensitivity high-frequency acoustic emission array is designed to enhance the ultrasound cranial-penetration signal intensity for cerebral blood-flow imaging based on encoded excitation technology to obtain brain neural information. Then, an automatically calibratable artificial intelligence pattern classifier is designed to decode the cerebral blood-flow signal and establish a quantitative relationship between it and the electroneurographic activity. Finally, noninvasive ultrasound neuromodulation and ion channel-switching technique are developed based on acoustic radiation force theory and acoustic tweezers technology **[**182–185**]**. We can activate mechanosensitive ion channels through ultrasound-radiation force by constructing a strong local gradient acoustic field and generating a modifiable acoustic-radiation force. We can then precisely control the excitability of neurons, apply ultrasound stimulation to specific nuclei and neural circuits, control specific sensations and behaviors of organisms, and realize a noninvasive closed-loop BCI. The implementation of this study is expected to provide new revolutionary tools in the fields of bioacoustics, health, rehabilitation, brain science, brain diseases, driverless vehicles, and artificial intelligence.
